# Supplementary material for: Moonlighting Arabidopsis molybdate transporter 2 family and GSH-complex formation facilitate molybdenum homeostasis
Source: Commun Biol. 2023 Aug 2;6:801. doi: 10.1038/s42003-023-05161-x (PMC10397214; doi:10.1038/s42003-023-05161-x)
Supplement: Supplementary file 2 — Supplementary Information [file 42003_2023_5161_MOESM2_ESM.pdf]

Supplementary

Moonlighting Arabidopsis molybdate transporter 2 family and GSH-complex formation facilitate molybdenum homeostasis

Jan-Niklas Weber, Rieke Minner-Meinen, Maria Behnecke, Rebekka Biedendieck, Veit G. Hänsch, Thomas W. Hercher, Christian Hertweck, Lena van den Hout, Lars Knüppel, Simon Sivov, Jutta Schulze, Ralf-R. Mendel, Robert Hänsch and David Kaufholdt

Supplementary Materials

Fig. S1: Split fluorescence channels of intracellular localization of MOT2-family members.

Fig. S2: Intracellular localization of MOT2-family members in *Arabidopsis* seedlings.

Fig. S3: Overview of histochemical GUS assay of WT control and *mot2:gus* lines.

Fig. S4: Impact of molybdate deprivation on *mot2*-KO survival and development.

Fig. S5: Impact of molybdate deprivation on macroscopic and molecular phenotype of *mot* multi-KOs.

Fig. S6: Abundance controls of BiFC experiments.

Fig. S7: Split-Luc assay of MOT2.1 and Cnx1 domains.

Tab. S1: Molybdate transport activity of MOT candidates tested in *Saccharomyces cerevisiae.*

Tab. S2: Two-way ANOVA analyses and Sidak post hoc tests for multiple comparison to analyze quantitative data in Figure 1C – Time to reach biomass index 40.

Tab. S3: Two-way ANOVA analyses and Sidak post hoc tests for multiple comparison to analyze quantitative data in Figure 3F – Fluorimetric GUS assay.

Tab. S4: One-way ANOVA analyses and Dunnet post hoc tests for multiple comparison to analyze quantitative data in Figure 4D – Molybdate uptake rate.

Tab. S5: Two-way ANOVA analyses and Tukey post hoc tests for multiple comparison to analyze quantitative data in Figure 4F – Nitrate reductase activity single KOs.

Tab. S6: List of generated vectors.

Tab. S7: List of used oligonucleotides.

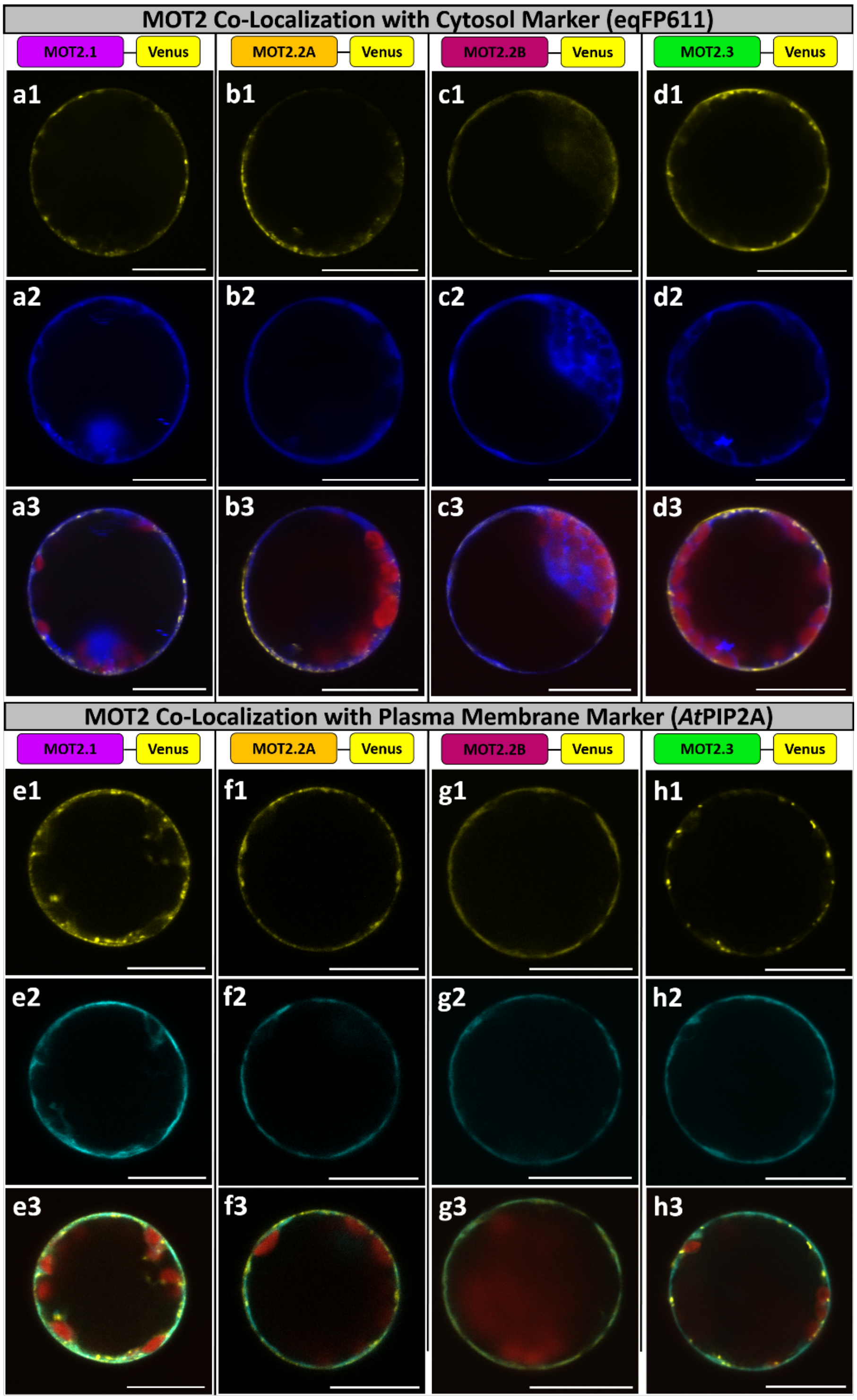

Fig. S1: Split fluorescence channels of intracellular localization of MOT2-family members. Transient chemical transformation of *N. benthamiana* mesophyll protoplasts with MOT2-Venus fusion constructs and co-expression of eqFP611 as cytosolic marker (**a-d**) or *At*TPIP2A-RFP as PM-marker (**e-h**). **a1-h1**: Venus detection channel shown in yellow. **a2-d2**: eqFP611 detection channel shown in blue. **e2-h2**: RFP detection channel shown in cyan. **a3-d3**: Merge of Venus detection channel, chloroplast detection channel (red) and eqFP611 detection channel as depicted in the main article in Fig 2 A-D. **e3-h3**: Merge of Venus detection channel, chloroplast detection channel (red) and RFP detection channel. Images were taken after 2-3 days using a C-Apochromat 40x/1.2 water immersion objective. Scale bars depict 20 µm.

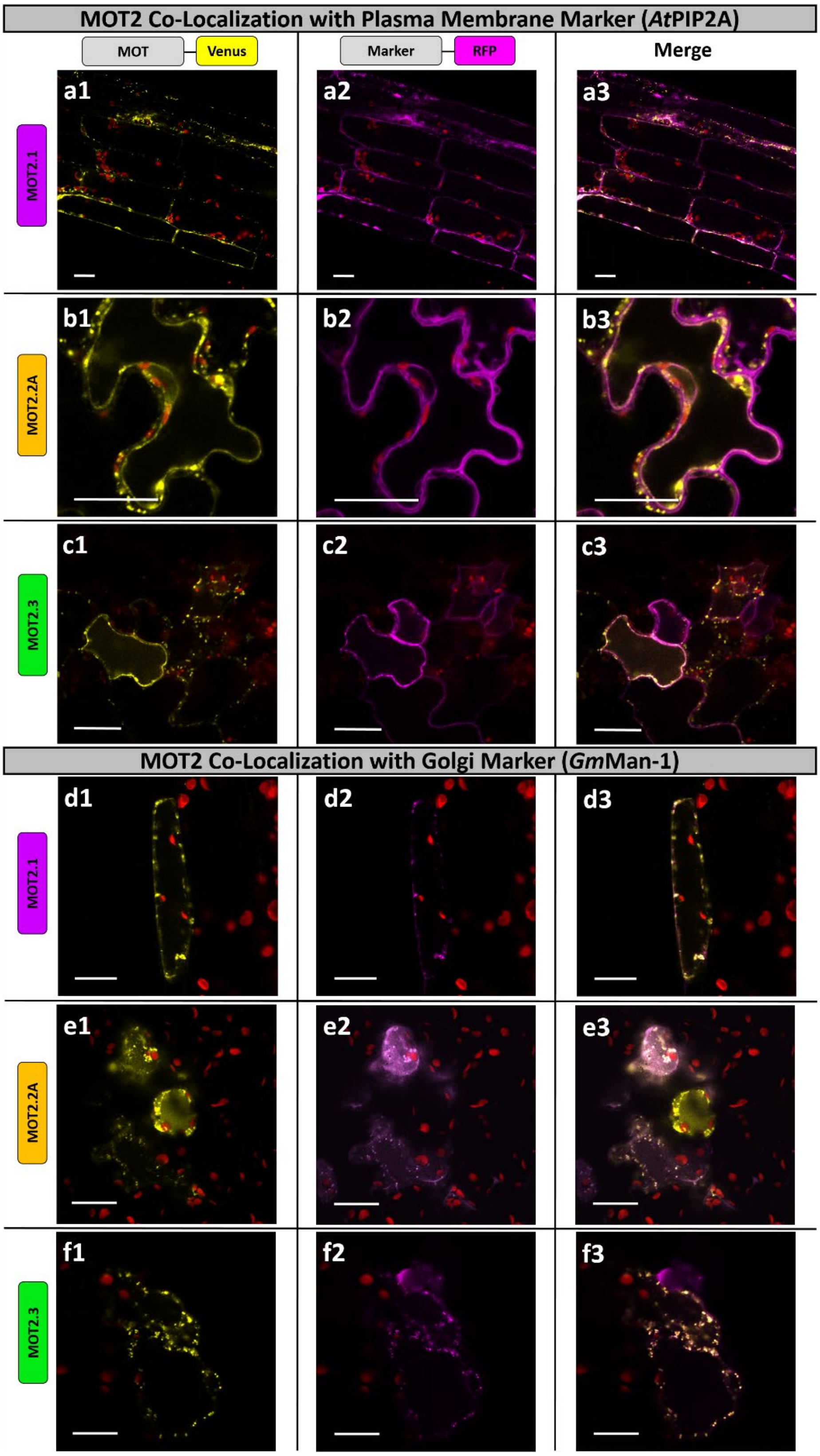

Fig. S2: Intracellular localization of MOT2-family members in *Arabidopsis* seedlings. a-c: FAST transformation of *A. thaliana* seedlings with MOT2-Venus constructs and *At*PIP2A -RFP as PM-marker. **d-f:** Co-localization with *Gm*Man-1-RFP as Golgi marker. Images show Venus signals (yellow; a1-f1), marker signals (magenta; a2-f2), and a merge (a3-f3). Chloroplast auto-fluorescence is shown in red. Images were taken after 2-3 days of incubation using a C-Apochromat 40x/1.2 water immersion objective. Scale bars depict 20 µm.

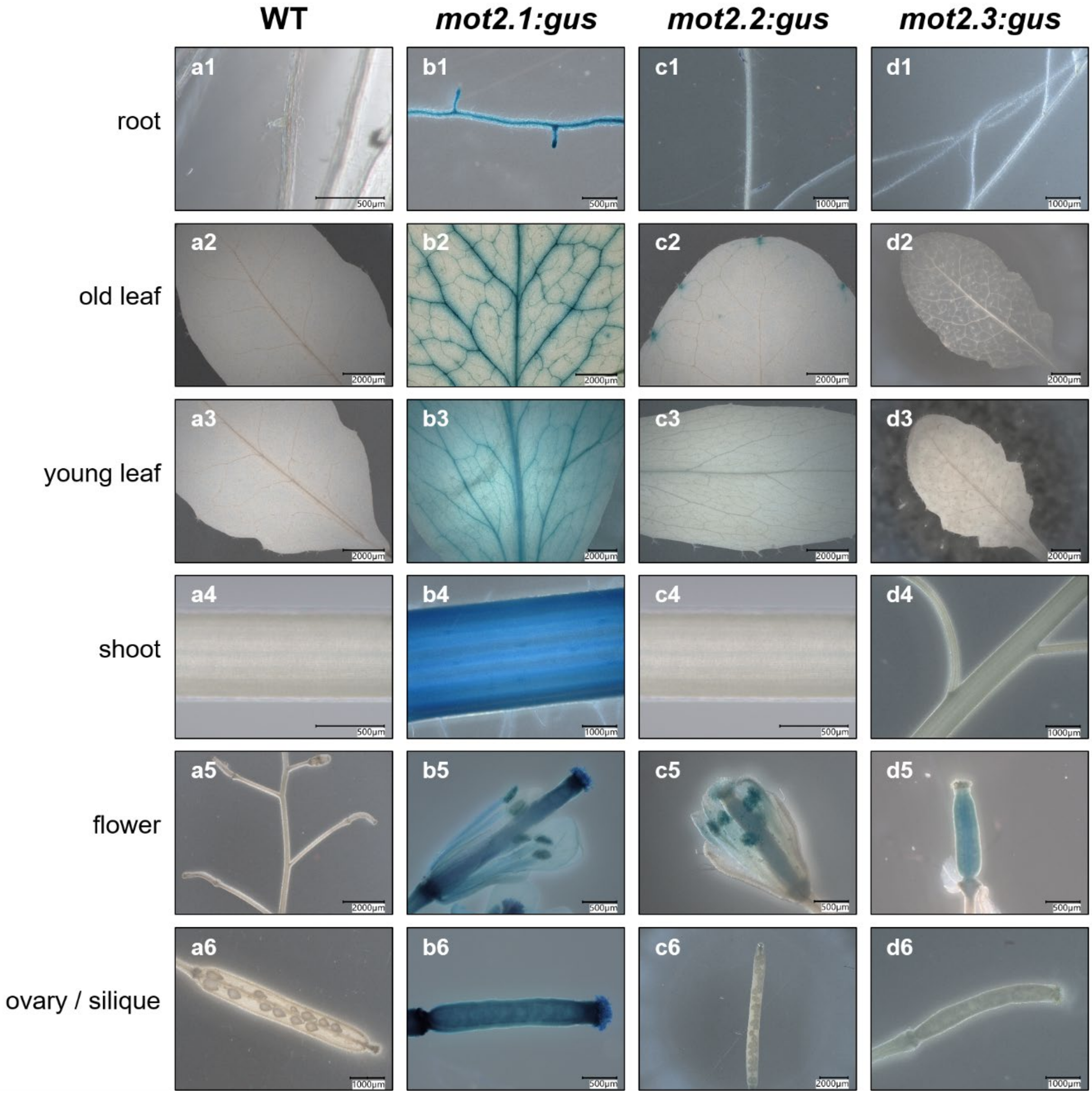

Fig. S3: Overview of histochemical GUS assay of WT control and *mot2:gus* lines. Histochemical GUS staining of (**a**) WT, (**b**) *mot2.1:gus*, (**c**) *mot2.2:gus*, and (**d**) *mot2.3:gus* lines. Depicted are root (1), old leaf (2), young leaf (3), shoot (4), flower (5) and ovary / silique (6). *A. thaliana* were grown hydroponically under +Mo conditions. Scale bars depict 500 - 2,000 µm as indicated in the panels.

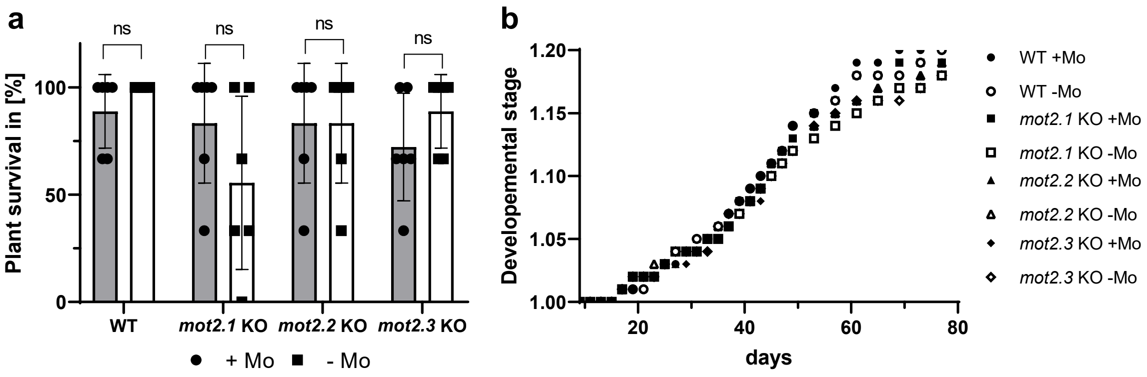

Fig. S4: Impact of molybdate deprivation on *mot2*-KO survival and development. a: Plant survival in percent of *mot2*-KO plants grown for 42 days. Columns depict the mean of 6 analyzed groups with 3 individuals each. Error bars depict standard deviation. Unpaired T-test was used for significance test. n.s. = not siginificant. **b**: Developmental stages according to Boyes *et al.* (2001) of *mot2*-KO plants grown for 81 days. Plants were grown hydroponically under molybdate availability (+Mo) and deprivation (-Mo).

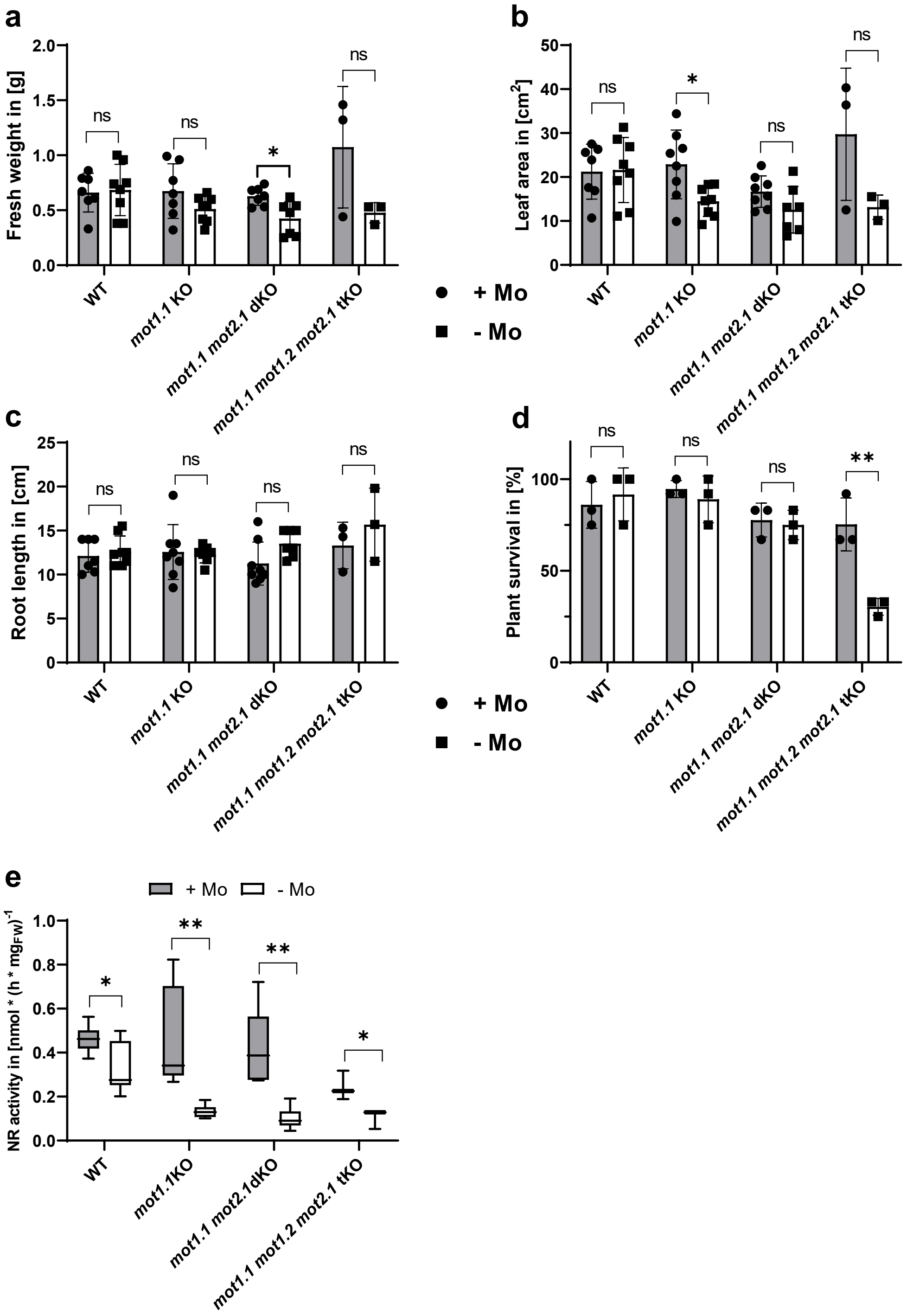

Fig. S5: Impact of molybdate deprivation on macroscopic and molecular phenotype of *mot* multi-KOs. *Arabidopsis mot1.1*-KO, *mot1.1 mot2.1* double-KO and *mot1.1 mot1.2 mot2.1* triple-KO were grown hydroponically under molybdate availability (+ / +Mo) and deprivation (– / –Mo) in a hydroponic system and harvested after 60 days. **a**: Fresh weight of rosette leaves. **b**: Leaf area of rosette leaves. **c**: Root length. **d**: Plant survival. **e**: NR activity of *mot* multi-KOs. Plotted is the mean of 3-9 individuals. Error bars depict standard deviation. Unpaired T-test was used for significance tests. ns = not significant, * = P ≤ 0.05, ** = P ≤ 0.01.

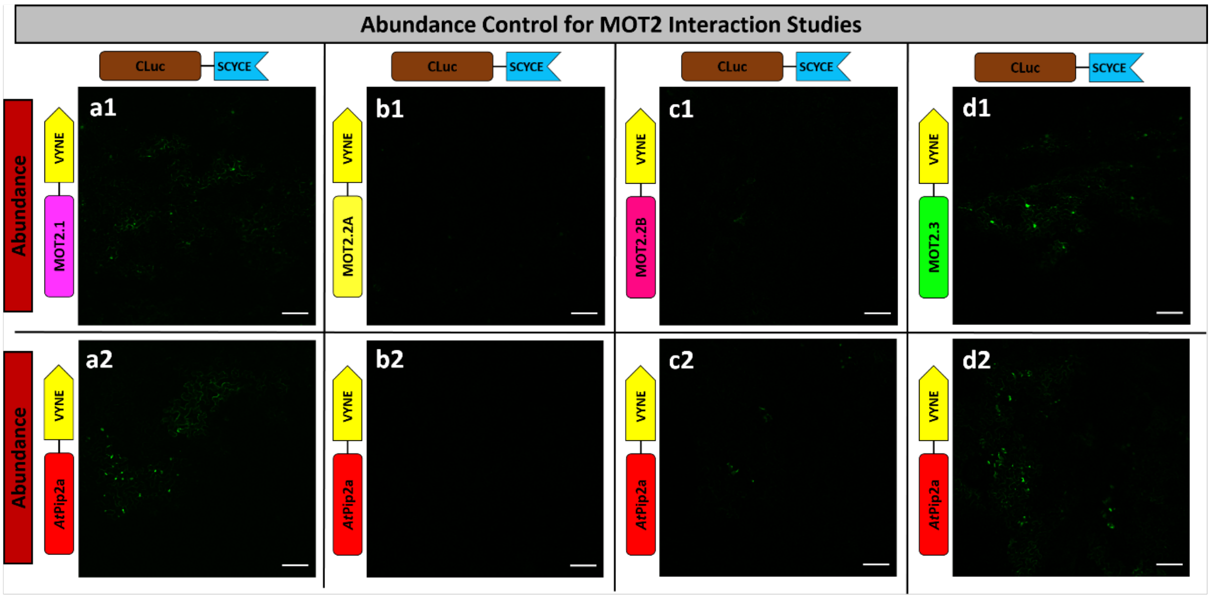

Fig. S6: Abundance controls of BiFC experiments. **a1-d1:** Transiently transformed leaves of *N. benthamiana* expressing MOT2-VYNE. **a2-d2:** Expression of *At*PIP2A-VYNE. Cnx1-SCYCE of BiFC approaches (Fig. 5) was replaced in both controls with cytosolic CLuc-SCYCE to display fluorescence intensities resulting from random interaction. Images were taken after 2-3 days of incubation with a Plan-Neofluar 10x/0.3 and scale bars depict 20 µm.

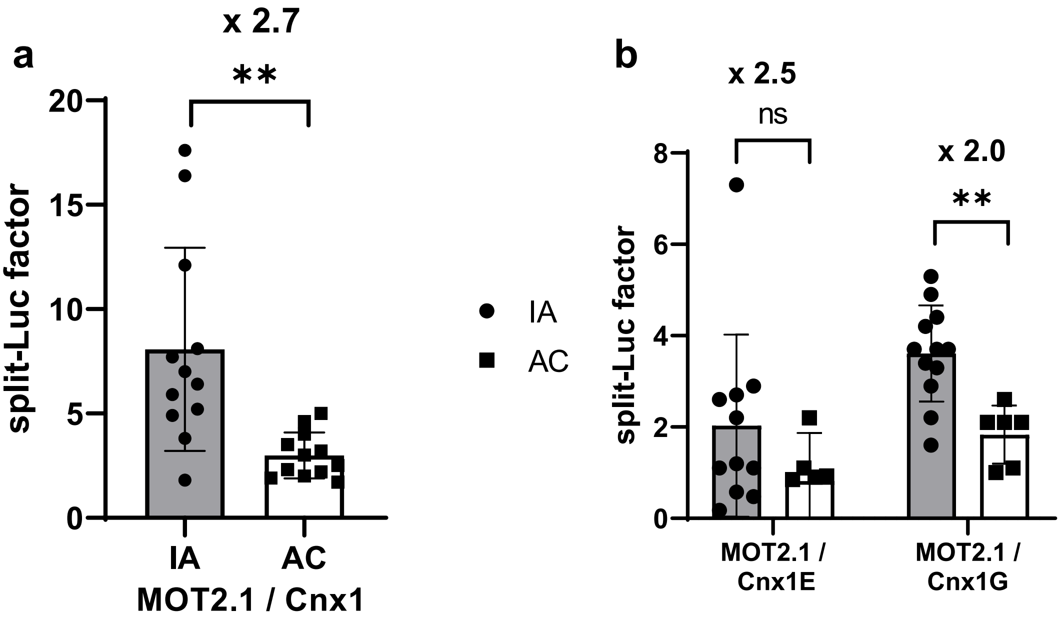

Fig. S7: Split-Luc assay of MOT2.1 and Cnx1 domains. a: Interaction approach (IA) of MOT2.1-CLuc and Cnx1-NLuc. Negative control consisted of *At*PIP2A-CLuc and Cnx1-NLuc and was used to calculate the IA split-Luc factor. Abundance control (AC) replaced Cnx1-NLuc with cytosolic SCYCE-NLuc and was used to calculate AC split-Luc factor. **b**: Split-Luc assay of MOT2.1 and Cnx1 E and G domain. Cytosolic NSP3-NLuc served as negative control and replaced Cnx1E-NLuc and Cnx1G-NLuc. CLuc-SCYCE was used as abundance control. Split-Luc factors were calculated accordingly. Factors calculated by dividing IA split-Luc factor and AC split-Luc factor are depicted above the columns. Columns show mean of 6-12 individuals. Error bars depict standard deviation. Unpaired T-test was used for significance tests. ns = not significant, ** = P ≤ 0.01.

Tab. S1: Molybdate transport activity of MOT candidates tested in *Saccharomyces cerevisiae*. *S. cerevisiae* strain YPH499[1] shows neither NR-activity nor presence of the Moco-precursor molybdopterin. Molybdopterin was measured by HPLC as its fluorescent derivative FormA by standard procedures[2]. NR-activity was measured according to Ringel *et al.*[3] and is given in nmol nitrite per µg protein per h. The complete Moco-biosynthesis pathway genes of *Neurospora crassa (nit*-1, *nit*-7, *nit*-8, *nit*-9, *nit*-12) and the gene for NR (*nit*-3) as characterized earlier[4,5] were used following the approach of Perli *et al.*[6] who had re-introduced the Moco-pathway and NR from a nitrate assimilating yeast into *S. cerevisiae*. Integration into *S. cerevisiae* strain YPH499 was performed using genomic integration according to Ishii *et al.*[7]. Additionally, *Arabidopsis* derived genes for *mot1* and *mot2* candidates were introduced into the yeast genome. Use of the *gal1* promoter allowed galactose induction of *mot2* gene expression. Incubation under galactose-induction and the presence of molybdate in the growth medium led to NR-activity as depicted as mean of three technical replicates ± standard deviation, whereas absence of molybdate abolished NR-activity.

Yeast Strain	Molybdopterin as determined by oxidation to FormA	NR-activity in [nmol $NO_2^- \cdot (\mu g \text{ protein} \cdot h)^{-1}$]
YPH499	-	0
+ Moco pathway	+	0
+ NR	-	0
+ Moco pathway + NR	+	0
+ Moco pathway + NR + *mot1.1*	+	1.5 ± 0.07
+ Moco pathway + NR + *mot1.2*	+	1.2 ± 0.18
+ Moco pathway + NR + *mot2.1*	+	0.12 ± 0.01
+ Moco pathway + NR + *mot2.2a*	+	0.38 ± 0.11
+ Moco pathway + NR + *mot2.2b*	+	0.40 ± 0.02
+ Moco pathway + NR + *mot2.3*	+	0.42 ± 0.03

References:

1. Sikorski, R. S. & Hieter, P. A System of Shuttle Vectors and Yeast Host Strains Designed for Efficient Manipulation of DNA in *Saccharomyces cerevisiae*. Genetics 122, 19-27 (1989).
2. Hercher, T. W. et al. Insights Into the Cnx1E Catalyzed MPT-AMP Hydrolysis. Bioscience Reports 40, BSR20191806 (2020).
3. Ringel, P. et al. Biochemical Characterization of Molybdenum Cofactor-free Nitrate Reductase from Neurospora crassa. Journal of Biological Chemistry 288, 14657–14671 (2013).
4. Probst, C. et al. Genetic Characterization of the *Neurospora crassa* Molybdenum Cofactor Biosynthesis. Fungal Genetics and Biology 66, 69–78 (2014).
5. Ringel, P. et al. Enzymatic Characterization of Recombinant Nitrate Reductase Expressed and Purified from Neurospora crassa. Fungal Genetics and Biology 80, 10–18 (2015).
6. Perli, T. et al. Engineering Heterologous Molybdenum-Cofactor-Biosynthesis and Nitrate-Assimilation Pathways Enables Nitrate Utilization by Saccharomyces cerevisiae. Metabolic Engineering 65, 11–29 (2021).
7. Ishii, J. et al. Microbial Fluorescence Sensing for Human Neurotensin Receptor Type 1 Using Gα-Engineered Yeast Cells. Analytical Biochemistry 446, 37–43 (2014).

Tab. S2: Two-way ANOVA analyses and Sidak post hoc tests for multiple comparison to analyze quantitative data in Figure 1C – Time to reach biomass index 40. Two-way ANOVA indicated significant differences between the induction status and the expressed *mot* candidate, respectively, with both P < 0.0001. Data sets were prior tested for normality using a Shapiro-Wilk test. The table contains the results of the conducted Sidak post-hoc test for multiple comparison of the mean of each group. **** = P < 0.0001

Tested combination	P-value	Significance level
mot1.1 +Gal vs. *mot1.1* –Gal	< 0.0001	****
mot1.2 +Gal vs. *mot1.2* –Gal	< 0.0001	****
mot2.1 +Gal vs. *mot2.1* –Gal	< 0.0001	****
mot2.2 +Gal vs. *mot2.2* –Gal	< 0.0001	****
mot2.3 +Gal vs. *mot2.3* –Gal	< 0.0001	****

Tab. S3: Two-way ANOVA analyses and Sidak post hoc tests for multiple comparison to analyze quantitative data in Figure 3F – Fluorimetric GUS assay. Two-way ANOVA indicated significant differences between the analyzed organ and the presence of molybdate, respectively, with P < 0.0001. The table contains the results of the conducted Tukey post-hoc test for multiple comparison of the mean of each group. ** = P ≤ 0.01, *** = P ≤ 0.001.

Tested combination	P-value	Significance level
root +Mo vs. root –Mo	0.0002	***
old leaf +Mo vs. old leaf –Mo	>0.9999	not significant
young leaf +Mo vs. young leaf –Mo	0.9999	not significant
shoot +Mo vs. shoot –Mo	0.0030	**
flower +Mo vs. flower –Mo	>0.9999	not significant
old leaf +Mo vs. young leaf +Mo	>0.9999	not significant
old leaf –Mo vs. young leaf –Mo	>0.9999	not significant

Tab. S4: One-way ANOVA analyses and Dunnet post hoc tests for multiple comparison to analyze quantitative data in Figure 4D – Molybdate uptake rate. One-way ANOVA indicated significant differences related to the presence of molybdate with $P \leq 0.0009$. The table contains the results of the conducted Dunnet post-hoc test for multiple comparison of the mean of each group. *** = $P \leq 0.001$.

Tested combination	P-value	Significance level
WT vs. *mot1.1* KO	0.0004	***
WT vs. *mot2.1* KO	0.1726	not significant

Tab. S5: Two-way ANOVA analyses and Tukey post hoc tests for multiple comparison to analyze quantitative data in Figure 4F – Nitrate reductase activity single KOs. Two-way ANOVA indicated significant influence of the molybdate presence with $P < 0.0001$, but no significant influence of the according genotype. The table contains the results of the conducted Tukey post-hoc test for multiple comparison of the mean of each group. ** = $P \leq 0.01$, *** = $P \leq 0.001$.

Tested combination	P-value	Significance level
WT +Mo vs. WT –Mo	0.2293	not significant
mot2.1 KO +Mo vs. *mot2.1* KO –Mo	0.9242	not significant
mot2.2 KO +Mo vs. *mot2.2* KO –Mo	0.0058	**
mot2.3 KO +Mo vs. *mot2.3* KO –Mo	0.0388	*
WT +Mo vs. *mot2.1* KO +Mo	0.3914	not significant

Tab. S6: List of generated vectors.

No.	Name	Reporter	Orientation	Resistance Bacteria / Plant	Source
1	pDONR/Zeo			Zeo / -	
2	pEntry-*mot2.1*-nostop			Zeo / -	
3	pEntry-*mot2.2a*-nostop			Zeo / -	
4	pEntry-*mot2.2b*-nostop			Zeo / -	
5	pEntry-*mot2.3*-nostop			Zeo / -	
6	pDest-*GW-venus*			Kan & Cmp / -	[43]
7	pExp-*mot2.1-venus*	Venus	C-terminus	Kan / -	
8	pExp-*mot2.2a-venus*	Venus	C-terminus	Kan / -	
9	pExp-*mot2.2b-venus*	Venus	C-terminus	Kan / -	
10	pExp-*mot2.3-venus*	Venus	C-terminus	Kan / -	
11	pExp-*eqFP611*	eqFP611		Spec / -	[47]
12	pEntry-*Atpip2a*			Zeo / -	
13	pDest-*GW-rfp*	RFP	C-terminus	Spec & Cmp / -	
14	pExp-*Atpip2a-rfp*	RFP	C-terminus	Spec / -	
15	pEntry-*Gmman-1*			Zeo / -	
16	pDest-*GW-scfp*	sCFP	C-terminus	Kan & Cmp / -	
17	pExp-Gm*man1-rfp*	RFP	C-terminus	Kan / -	
18	pDest-*gfp11-GW*	GFP11	N-terminus	Spec & Cmp / -	
19	pDest-*GW-gfp11*	GFP11	C-terminus	Spec & Cmp / -	
20	pExp-*gfp11-mot2.1*	GFP11	N-terminus	Spec / -	
21	pExp-*mot2.1-gfp11*	GFP11	C-terminus	Spec / -	
22	pExp-*gfp11-mot2.2a*	GFP11	N-terminus	Spec / -	
23	pExp-*mot2.2a-gfp11*	GFP11	C-terminus	Spec / -	
24	pExp-*gfp11-mot2.2b*	GFP11	N-terminus	Spec / -	
25	pExp-*mot2.2b-gfp11*	GFP11	C-terminus	Spec / -	
26	pExp-*gfp11-mot2.3*	GFP11	N-terminus	Spec / -	
27	pExp-*mot2.3-gfp11*	GFP11	C-terminus	Spec / -	
28	pExp-*gfp1-10*	GFP1-10		Spec / -	
29	pExp-*SP-gfp1-10*	GFP1-10		Spec / -	
30	pEntry-*mot2.1_endoPro*			Zeo / -	
31	pEntry-*mot2.2_endoPro*			Zeo / -	
32	pEntry-*mot2.3_endoPro*			Zeo / -	
33	pDest-*GW-gfp-gus*	GFP-GUS		Spec & Cmp / Kan	
34	pExp-*mot2.1:gpf-gus*	GFP-GUS		Spec / Kan	
35	pExp-*mot2.2:gpf-gus*	GFP-GUS		Spec / Kan	
36	pExp-*mot2.3:gpf-gus*	GFP-GUS		Spec / Kan	
37	pDest-*GW-vyne*	VYNE	C-terminus	Kan & Cmp / -	[46]
38	pDest-*GW-scyce*	SCYCE	C-terminus	Kan & Cmp / -	[46]
39	pExp-*mot2.1-vyne*	VYNE	C-terminus	Kan / -	
40	pExp-*mot2.2a-vyne*	VYNE	C-terminus	Kan / -	
41	pExp-*mot2.2b-vyne*	VYNE	C-terminus	Kan / -	
42	pExp-*mot2.3-vyne*	VYNE	C-terminus	Kan / -	
43	pExp-*Atpip2a-vyne*	VYNE	C-terminus	Kan / -	
44	pExp-*cnx1-scyce*	SCYCE	C-terminus	Kan / -	
45	pExp-*cluc-scyce*	SCYCE	C-terminus	Kan / -	
46	pDest-GW-cluc	CLuc	C-terminus	Kan & Cmp / -	[45]
47	pExp-*mot2.1-cluc*	CLuc	C-terminus	Kan / -	
48	pExp-*Atpip2-cluc*	CLuc	C-terminus	Kan / -	[45]
49	pExp-*cnx1-nluc*	NLuc	C-terminus	Kan / -	[45]
50	pExp-*cnx1e-nluc*	NLuc	C-terminus	Kan / -	[45]
51	pExp-*cnx1g-nluc*	NLuc	C-terminus	Kan / -	[45]
52	pExp-*nsp3-nluc*	NLuc	C-terminus	Kan / -	[45]

Tab. S7: List of used oligonucleotides.

No.	Primer name	Sequence	Purpose
1	attB1_MOT2.1_for	GGGGACAAGTTTGTACAAAAAAGCAGGCTTAA CCATGGAGATTTTCTACTACTTGG	Cloning of *mot2* CDS
2	MOT2.1-nostop_attB2_rev	GGGGACCACTTTGTACAAGAAAGCTGGGTCTAT GTTGAGGGGATCTTCTTCTG	Cloning of *mot2* CDS
3	attB1_MOT2.2A_for	GGGGACAAGTTTGTACAAAAAAGCAGGCTTAA CCATGGAGATCTTCTACTTCGTGGTGTTC	Cloning of *mot2* CDS
4	attB1_MOT2.2B_for	GGGGACAAGTTTGTACAAAAAAGCAGGCTTAA CCATGAATTTATGTCCCTGGCGGC	Cloning of *mot2* CDS
5	MOT2.2-nostop_ attB2_rev	GGGGACCACTTTGTACAAGAAAGCTGGGTCTG GGTTTAGAGGGTCAGCTTCTG	Cloning of *mot2* CDS
6	attB1_MOT2.3_for	GGGGACAAGTTTGTACAAAAAAGCAGGCTTAA CCATGGAGGTTTTCTACTACTTGGTGTTT	Cloning of *mot2* CDS
7	MOT2.3-nostop_ attB2_rev	GGGGACCACTTTGTACAAGAAAGCTGGGTCGA GGGTAAGAGGATCAACTTCCG	Cloning of *mot2* CDS
8	attB1_AtPIP2A_for	GGGGACAAGTTTGTACAAAAAAGCAGGCTTAA CCATGGCAAAGGATGTGGAAGCCG	Cloning of *pip2* CDS
9	AtPIP2A-nostop_ attB2_rev	GGGGACCACTTTGTACAAGAAAGCTGGGTCGA CGTTGGCAGCACTTCTGAATG	Cloning of *pip2* CDS
10	GmMan1_t-TM_1_for	ATGGCTAGCGGGAGCAGATCAGTGGGTAGCAG CAGCAGCAAATGGAGGTACTGCAACCCTACTAG TTACTTGAAGCGCCCAAAGCGTCT	Cloning of *man-1* CDS
11	GmMan1_t-TM_2_rev	GTCAGTTTGACGGTCCCAGAAAACGAAAGAGA CACAAACGAAAACGATGAAGAGCAGAGCAAGA CGCTTTGGGCGCTTCAAGTAACTAGTA	Cloning of *man-1* CDS
12	attB1_GmMan1_t-TM_for	GGGGACAAGTTTGTACAAAAAAGCAGGCTTAA CCATGGCTAGCGGGAGCAGATCAGTGGGTAG	Cloning of *man-1* CDS
13	GmMan1_t-TM-nostop_ attB2_rev	GGGGACAAGTTTGTACAAAAAAGCAGGCTTAA CCATGAAGGTACAGGAGGGTTTG	Cloning of *man-1* CDS
14	attB1_endoMOT2.1_-1998_for	GGGGACAAGTTTGTACAAAAAAGCAGGCTTACC GATGGGAGATTGCACCTTAC	Cloning of *mot2.1* promoter
15	endoMOT2.1_attB2_rev	GGGGACCACTTTGTACAAGAAAGCTGGGTCTGT CGTTCGATCCAGAAACTATC	Cloning of *mot2.1* promoter
16	attB1_endoMOT2.2_-1924_for	GGGGACAAGTTTGTACAAAAAAGCAGGCTTAG TCTACATACAGATTCATAAAGC	Cloning of *mot2.2* promoter
17	endoMOT2.2_attB2_rev	GGGGACCACTTTGTACAAGAAAGCTGGGTCTGA TACGATCAGATCTCGCAAGG	Cloning of *mot2.2* promoter

18	attB1_endoMOT2.3_-1980_for	GGGGACAAGTTTGTACAAAAAAGCAGGCTTAG TCTTGTAGCATTATGGATTGGA	Cloning of *mot2.3* promoter
19	endoMOT2.3_attB2_rev	GGGGACCACTTTGTACAAGAAAGCTGGGTCTGT TGCTTCTCTCTCACTCTCTC	Cloning of *mot2.3* promoter
20	MOT2.1_genomic_114-134_for	TGCACTTAACACACGGTGGA	Genotyping
21	PAC161_LB_rev	ATATTGACCATCATACTCATTGC	Genotyping
22	MOT2.1_genomic_200-221_rev	CCAGATCGGATCGAAACAGGA	Genotyping
23	MOT2.2_genomic_737-756_for	ATTAGGGGTCGCAAGAGAGC	Genotyping
24	MOT2.2_genomic_1230-1252_rev	GTGGAAGAATAAAGCTGGAAGCA	Genotyping
25	pROK2_101-121_rev	AATCAGCTGTTGCCCGTCTC	Genotyping
26	mot2.3_geno_1545-1566_for	GCAATCTGTAGACAAGGTTGGA	Genotyping
27	mot2.3_geno_1987-2007_rev	TGCAGGATGGGATGAATGGAG	Genotyping
