# Supplementary material for: Moonlighting Arabidopsis molybdate transporter 2 family and GSH-complex formation facilitate molybdenum homeostasis
Source: Commun Biol. 2023 Aug 2;6:801. doi: 10.1038/s42003-023-05161-x (PMC10397214; doi:10.1038/s42003-023-05161-x)
Supplement: Supplementary file 3 — Description of Additional Supplementary Files [file 42003_2023_5161_MOESM3_ESM.pdf]

Description of Additional Supplementary Files

File name: Supplementary Data 1

Description: The numeric data used to generate graphs in the present work.
